# Prognostic and clinicopathological value of Gli-1 expression in gastric cancer: A meta-analysis

**DOI:** 10.18632/oncotarget.12011

**Published:** 2016-09-13

**Authors:** Li Lu, Menglin Wu, Feixiang Zhao, Weihua Fu, Weidong Li, Xue Li, Tong Liu

**Affiliations:** ^1^ Department of General Surgery, Tianjin Medical University General Hospital, Tianjin, China; ^2^ Department of Radiology, Second Hospital of Tianjin Medical University, Tianjin, China

**Keywords:** Gli-1, gastric cancer, prognosis

## Abstract

Glioma associated oncogene-1 (Gli-1) is considered as a strong positive activator of downstream target genes of hedgehog signal pathway in mammalians. However, its diagnostic and prognostic value in gastric cancer remains unclear and controversial. Therefore, a quantitative meta-analysis was conducted to determine the clinical value of Gli-1 in gastric cancer patients. Twelve eligible articles with 886 gastric cancer patients were included in this meta-analysis. The relationship between Gli-1 expression in gastric cancer patients and clinicopathological features and 5-year overall survival (OS) was evaluated using pooled odds ratios (ORs) and hazard ratio (HR) with 95% confidence intervals (CIs). The meta-analysis showed that the upregulated Gli-1 was associated with sample type (gastric cancer tissues) (OR 10.31, 95%CI 7.14-14.88; *P* = 0.000), differentiation type (OR 3.76, 95%CI 2.55-5.53; *P* = 0.000), depth of invasion (OR 8.17, 95%CI 3.60-18.55; *P* = 0.000), lymph node metastasis (OR 3.97, 95%CI 2.73-5.78; *P* = 0.000) and high TNM stage (OR 3.65, 95%CI 1.89-7.04; *P* = 0.000). Three studies including 316 patients were assessed for the correlation between Gli-1 and 5-year OS, which indicated that positive Gli-1 expression was associated with poor prognosis in gastric cancer patients (HR 2.14, 95%CI 1.35-3.40; *P* = 0.001). Little publication bias was identified by funnel plots and Egger's tests. The sensitivity analysis indicated that no study substantially influenced pooled OR/HR. Taken together, Gli-1 is a credible indicator for highly aggressive tumor with poor prognosis in gastric cancer patients.

## INTRODUCTION

In recent years, gastric cancer has become the third most common malignancy in China [1]. And it is the third highest leading cause of cancer-related mortality in the world [2]. Although surgical methods and chemotherapeutic regimens have made progressing development, which have improved the prognosis of gastric cancer patients to some extent, the 5-year survival rate is still below 35%. In China, because the symptoms of early gastric cancer are nonspecific, most of patients are diagnosed with gastric cancer at an advanced stage and with poor prognosis. Moreover, some patients are unsuitable for radical surgery because of the intraperitoneal or distant metastasis, which further reduces survival rate. In other words, gastric carcinomas with high potential of invasion or metastasis have made serious challenges for patients. Thus, a better understanding of the mechanisms driving the invasion and metastasis of gastric cancer is crucial. It is vital to identify a critical biomarker which can point out more aggressive tumors, meanwhile, this specific biomarker can serve as a novel therapeutic target.

The process of metastasis is complex and involves the spread of carcinoma cells from the primary site to distant sites. Epithelial-to-mesenchymal transition (EMT) is an essential event in the initial step of the metastatic cascade. Phenotype changes have been reported in epithelial carcinoma cells during EMT, including the loss of cell-cell contacts, cell polarity, epithelial markers (especially E-cadherin), and overexpression of mesenchymal markers (such as vimentin and N-cadherin) [3]. Meanwhile, changes in the cytoskeleton enhance carcinoma cells' abilities of invasiveness and motility [4-5].

The Hh signaling pathway is considered as a vital pathway in embryonic growth and differentiation, the preservation of stem cells and tumorigenesis [6-7], which also has been proved to have a close association with EMT [8]. Previous research reported that glioma associated oncogene-1 (Gli-1) exhibited a strong positive activating effect of downstream target genes of the Hh pathway [9]. Besides, several studies have found that Gli-1 can induce the expression of Snail. As one of the transcriptional regulators of EMT, Snail can decrease the expression of E-cadherin while increase the expression of N-cadherin [10-11]. These results suggest that Gli-1 may have a close relationship with the process of EMT by abnormally activating Snail. In consideration of the relationship between tumor metastasis and EMT, the abnormal activation of Gli-1 may contribute to a potential high malignancy degree of cancer. In consensus with the above hypothesis, Gli-1 exhibited a significant correlation with the tumor migration in pancreatic cancer [12]. In esophageal cancer, Gli-1 was also identified as a strong and independent prognostic factor for poor outcome [13].

However, the clinical evidence of the relationship between Gli-1 and tumor invasion or prognosis in gastric cancer is insufficient at present. Hence, a meta-analysis of published data was performed to systematically elucidate whether Gli-1 overexpression would have correlation with the tumorigenesis and prognosis in patients with gastric cancer.

## RESULTS

### Identification of eligible studies

**Figure 1:**
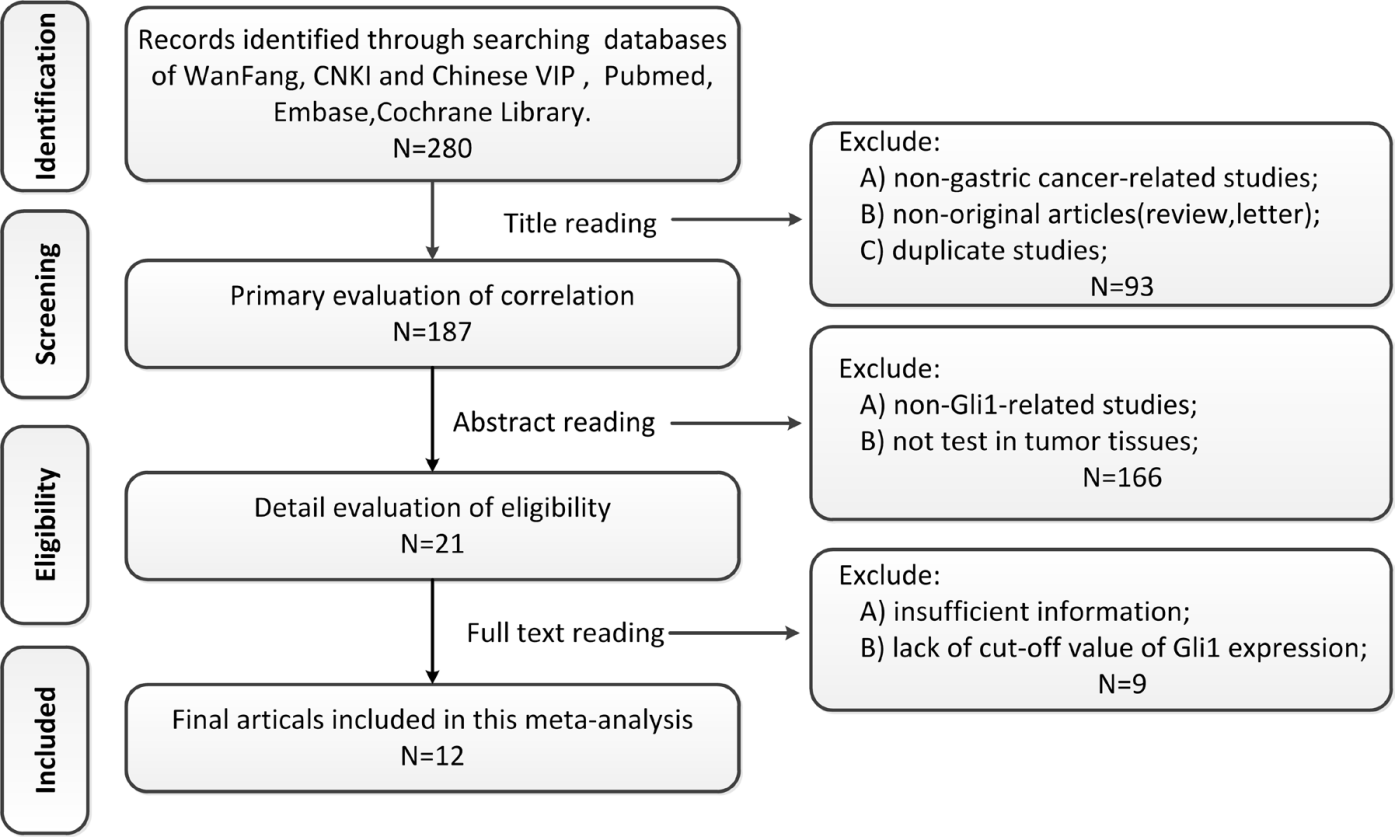
Flow chat of study selection

Firstly, a total of 280 potential related studies were selected from the databases on the basis of our defined criteria. Endnote, the literature manager software, was utilized to exclude non-gastric-cancer-studies, non-original articles (review, letter) and the duplicated studies (*n* = 93) through reading titles. The remaining 187 articles were further assessed by screening the abstracts, among which 166 articles were excluded due to non-Gli-1-related human studies, not test in tumor tissues. A total of 21 studies were assessed by reading the full texts, and then 9 studies were excluded due to insufficient information and/or lack of cut-off value of Gli-1 expression. Eventually, 12 eligible articles with 886 gastric cancer patients were included in this meta-analysis. Detailed selection process was illustrated in a flow chart (Figure 1).

### Study characteristics and quality assessment

Ten of eligible studies used the immunological histological chemistry (IHC) method to evaluate the expression of Gli-1 in gastric cancer tissues, the rest 2 studies used *in situ* hybridization (ISH) method. All of the studies were conducted in China. The publication years of all studies ranged from 2005 to 2016. The sample-size ranged from 20 to 121, and the percentage of positive Gli-1 expression ranged from 53.2% to 88.0%. The NOS scores varied from 7 to 9, which indicated that the quality of all studies was high. Further detailed characteristics were listed in Tables 1-2.

**Table 1:** Characteristics of included studies

No. of Studies	Author	Year	Country	Cases (n)	Method	Antibody Dilution	Cut-off Value	Positive Percentage
1	Chen[14]	2016	China	101	IHC	1:100	5 score	57.8%
2	Zhang[15]	2016	China	94	IHC	NA	10%	53.2%
3	Yang[16]	2015	China	20	ISH	-	-	55.0%
4	Zheng[17]	2014	China	98	IHC	NA	50%	77.6%
5	Qi[18]	2014	China	96	IHC	NA	2 score	68.8%
6	Liu[19]	2014	China	65	IHC	1:200	3 score	64.5%
7	Wang[20]	2014	China	121	IHC	1:200	10%	79.3%
8	Yan[21]	2013	China	50	IHC	1:500	3 score	88.0%
9	Feng[22]	2012	China	70	IHC	NA	2 score	74.3%
10	Ouyang[23]	2011	China	54	IHC	1:50	10%	61.1%
11	Rong[24]	2006	China	85	IHC	1:150	0%	75.3%
12	Ma[25]	2005	China	32	ISH	-	-	68.8%

Abbreviations: IHC = immunohistochemistry, ISH= in situ hybridization, NA= not available.

**Table 2:** Newcastle-Ottawa Quality Assessment Scale of included studies

Study	Selection				Comparability	Outcome			Score
	Representativeness of Exposed Cohort^1^	Selection of Nonexposed Group^2^	Ascertainment of Exposure^3^	Outcome of Interest^4^	Comparability of Cohorts^5^	Assessment of Outcome^6^	Length of Follow-up^7^	Adequacy of Follow-up^8^	
Chen[14]	1	1	1	1	1	1	1	1	8
Zhang[15]	1	1	0	1	2	1	1	0	7
Yang [16]	1	0	1	1	1	1	1	1	7
Zheng [17]	1	1	1	1	2	1	1	1	9
Qi [18]	1	1	1	1	2	1	1	1	9
Liu [19]	1	1	1	1	2	1	0	1	8
Wang[20]	1	1	1	1	2	1	1	1	9
Yan[21]	1	1	1	1	2	1	0	0	7
Feng[22]	1	1	1	1	2	1	1	1	9
Ouyang[23]	1	1	1	1	2	1	0	1	8
Rong [24]	1	1	1	1	2	1	0	1	8
Ma[25]	1	1	1	1	2	1	0	1	8

Score was achieved for each item if.

1. The exposed cohort truly or somewhat represented the average in the community.

2. The non-exposed cohort was drawn from the same community as the exposed cohort.

3. Ascertainment of exposure was secure record or structured interview.

4. Outcome of interest was not present at start of study.

5. Study controls for the most important factor.

6. Assessment of outcome was from independent blind assessment or record linkage.

7. Follow-up was long enough for outcomes to occur.

8. No subject lost to follow-up or subjects lost to follow-up unlikely to introduce bias or description provided of those lost.

### Association between Gli-1 in gastric cancer and clinicopathological features

**Figure 2:**
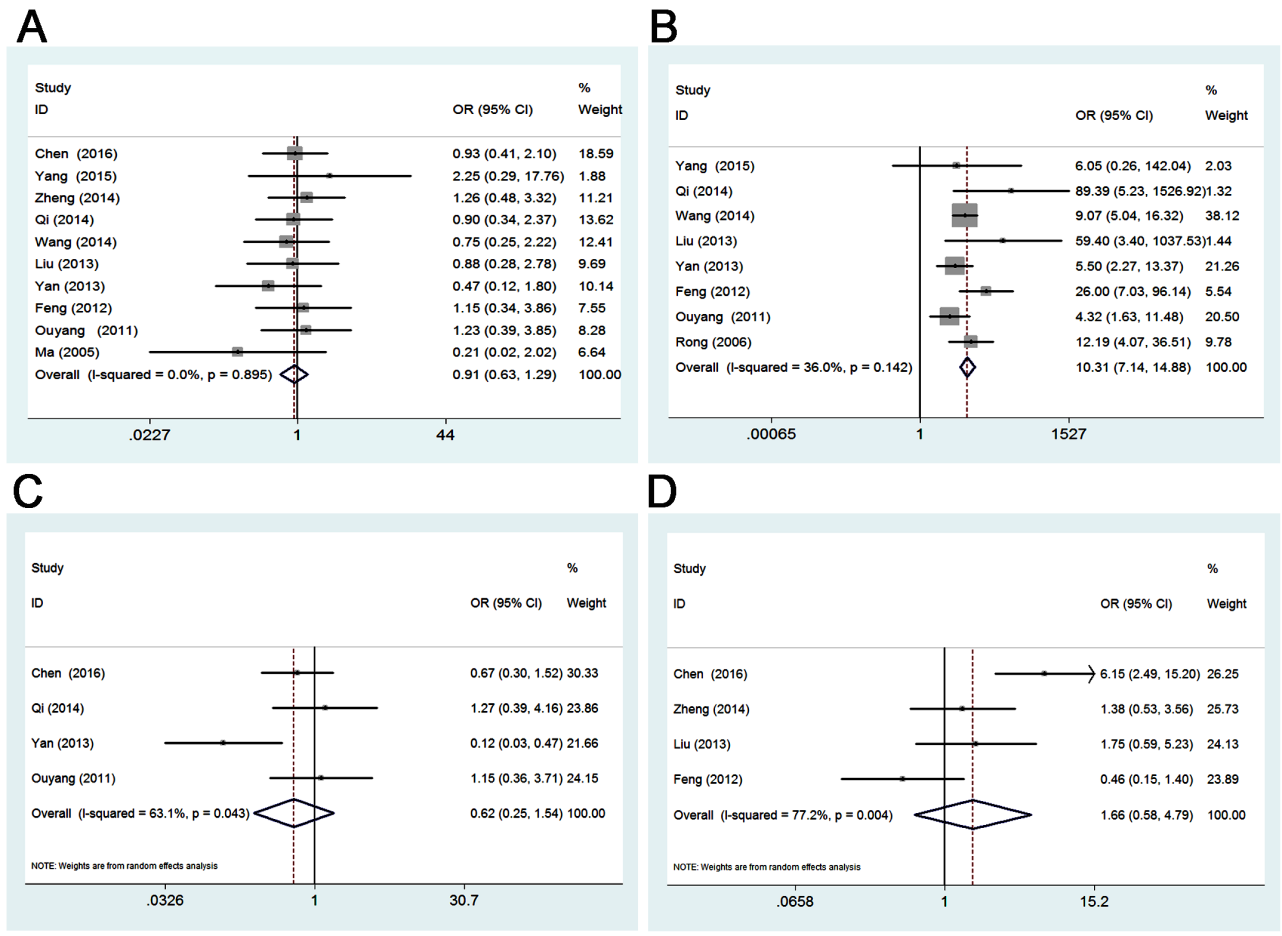
**A.** **Forest plot of studies evaluating the relationship between Gli-1 expression and gender**. **B.** Forest plot of studies evaluating the relationship between Gli-1 expression and sample type. **C.** Forest plot of studies evaluating the relationship between Gli-1 expression and tumor location. **D.** Forest plot of studies evaluating the relationship between Gli-1 expression and tumor size.

**Figure 3:**
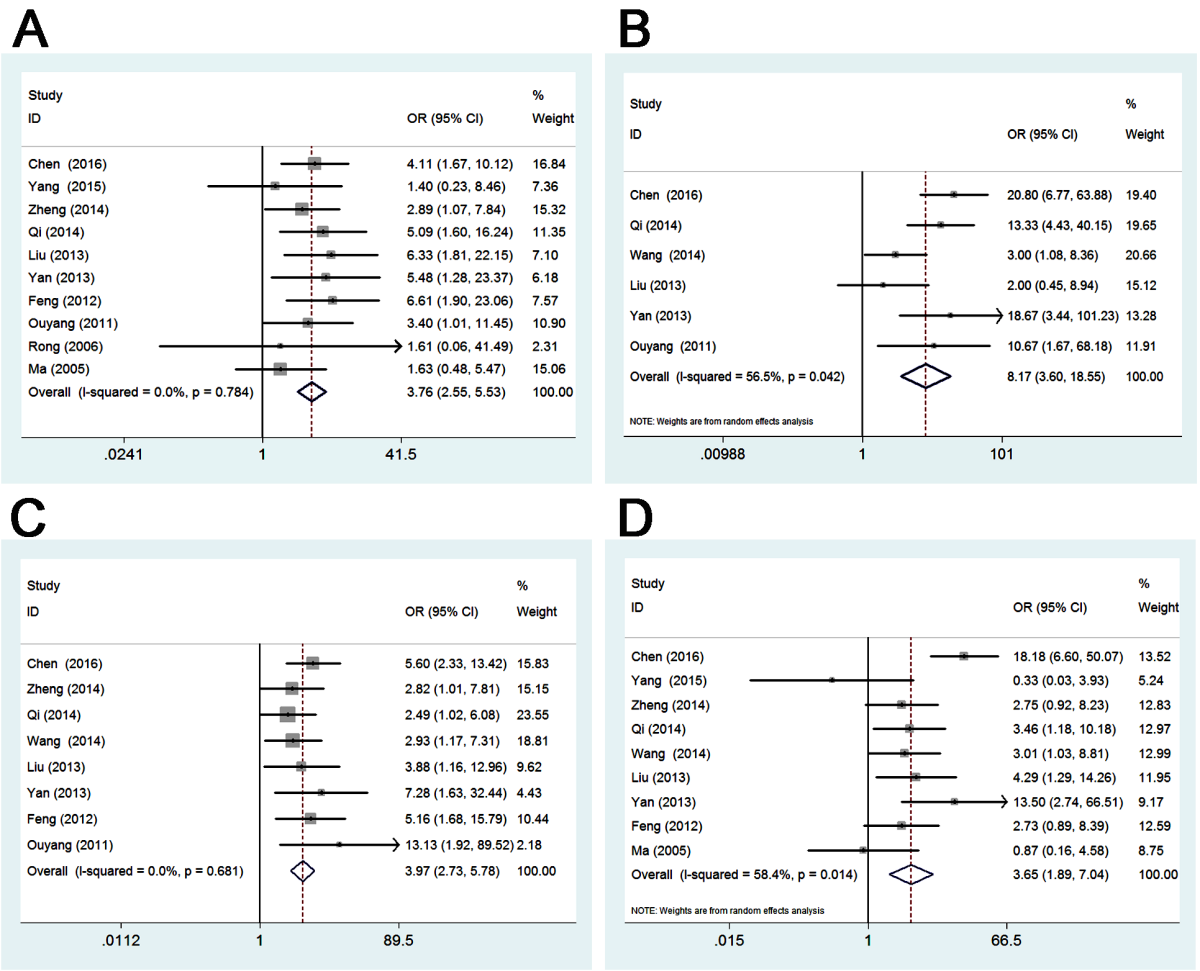
**A.** **Forest plot of studies evaluating the relationship between Gli-1 expression and differentiation type**. **B.** Forest plot of studies evaluating the relationship between Gli-1 expression and depth of invasion. **C.** Forest plot of studies evaluating the relationship between Gli-1 expression and lymph node metastasis. **D.** Forest plot of studies evaluating the relationship between Gli-1 expression and TNM.

To confirm the clinical value of Gli-1, the correlations between Gli-1 and numerous clinicopathological parameters were explored precisely. As seen in Table 3 and Figures 2-3, pooled ORs of 12 eligible studies showed the upregulated Gli-1 was associated with sample type (OR 10.31, 95%CI 7.14-14.88; *P* = 0.000), differentiation type (OR 3.76, 95%CI 2.55-5.53; *P* = 0.000), depth of invasion (OR 8.17, 95%CI 3.60-18.55; *P* = 0.000), lymph node metastasis (OR 3.97, 95%CI 2.73-5.78; *P* = 0.000), high TNM stage (OR 3.65, 95%CI 1.89-7.04; *P* = 0.000). However, no relationship was found between positive Gli-1 expression and gender (OR 0.91, 95%CI 0.63-1.29; *P* = 0.588), tumor location (OR 0.62, 95%CI 0.25-1.54; *P* = 0.298), or tumor size (OR 1.66, 95%CI 0.58-4.79; *P* = 0.346).

**Table 3:** Main results for meta-analysis between Gli-1 and clinicopathological features/overall survival (OS) and publication bias (Egger's test)

Correlation between Gli-1 and clinicopathological features/OS	No. of studies	Overall OR/HR (95%CI)	*z*, *P*_OR/HR_	Heterogeneity test (*I*^2^, *P*_bias_)	Publication bias (Egger's test) (*t*, *P*_publication bias_)
Gender (male vs. female)	1, 3, 4, 5, 6, 7, 8, 9, 10, 12	0.91 (0.63, 1.29)	0.54, 0.588	0.0%, 0.895	−0.80, 0.447
Sample type (gastric cancer tissues vs. normal gastric tissues)	3, 5, 6, 7, 8, 9, 10, 11	10.31 (7.14, 14.88)	12.44, 0.000	36.0%, 0.142	1.48, 0.189
Tumor location (antrum vs. non-antrum)	1, 5, 8, 10	0.62 (0.25, 1.54)	1.04, 0.298	63.1%, 0.043	−0.41, 0.720
Tumor size (≥5cm vs. <5cm)	1, 4, 6, 9	1.66 (0.58, 4.79)	0.94, 0.346	77.2%, 0.004	−1.77, 0.218
Differentiation type (poor/undifferentiated vs. well/moderate)	1, 3, 4, 5, 6, 8, 9, 10, 11, 12	3.76 (2.55, 5.53)	6.70, 0.000	0.0%, 0.784	−0.43, 0.683
Depth of invasion (T3/T4 vs. T1/T2)	1, 5, 6, 7, 8, 10	8.17 (3.60, 18.55)	5.02, 0.000	56.5%, 0.042	0.18, 0.868
Lymph node metastasis (Yes vs. No)	1, 4, 5, 6, 7, 8, 9, 10	3.97 (2.73, 5.78)	7.20, 0.000	0.0%, 0.681	2.28, 0.063
TNM (III/IV vs. I/II)	1, 3, 4, 5, 6, 7, 8, 9, 12	3.65 (1.89, 7.04)	3.86, 0.000	58.4%, 0.014	−1.49, 0.179
5-year OS	1, 2, 7	2.14 (1.35, 3.40)	3.23, 0.001	39.2%, 0.193	1.18, 0.448

Abbreviations: HR=hazard ratio, OR = odds ratio, OS =overall survival.

### Association between Gli-1 in gastric cancer and 5-year overall survival

**Figure 4:**
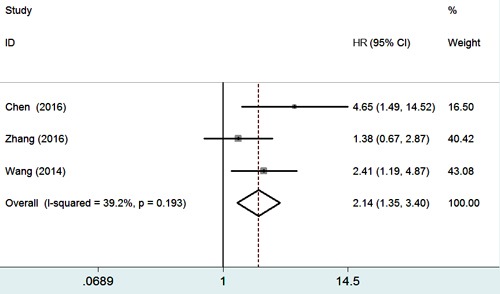
Forest plot of studies evaluating the relationship between Gli-1 expression and 5-year overall survival

Three studies including 316 patients were assessed for the correlation between Gli-1 and 5-year overall survival (OS). The result (Table 3 and Figure 4) indicated that positive Gli-1 expression was associated with poor prognosis in gastric cancer patients (HR 2.14, 95%CI 1.35-3.40; *P* = 0.001).

### Publication bias and sensitivity analysis

**Figure 5:**
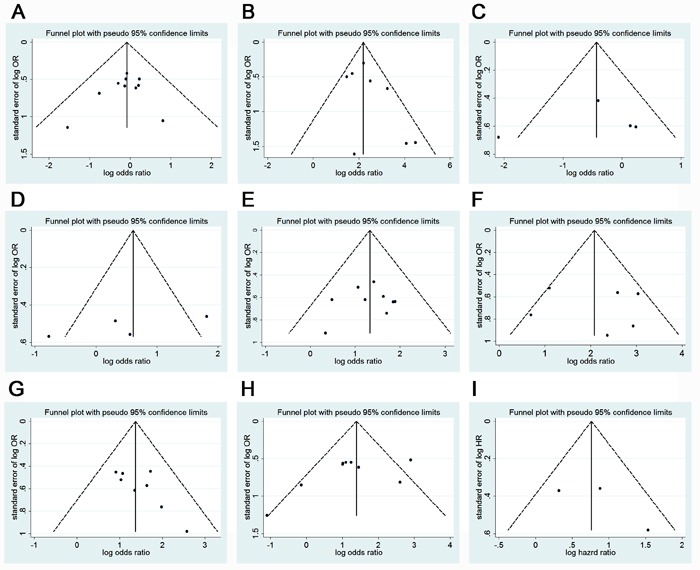
Funnel plot for publication bias test of Gli-1 related studies **A.**, Gender; **B.**, Sample type; **C.**, Tumor location; **D.**, Tumor size; **E.**, Differentiation type; **F.**, Depth of invasion; **G.**, Lymph node metastasis; **H.**, TNM; **I.**, 5-year overall survival.

In our meta-analysis, funnel plots as well as Egger's tests were introduced to examine potential publication bias. A funnel plot of every 2 groups was conducted with OR/HR as the x-axis and stand error (SE) of log OR/HR as the y-axis, respectively. All of the plots were symmetric, indicating that publication bias was low (Figure 5). In accordance with the results of funnel plots, little publication bias was identified by Egger's tests (Table 3).

Sensitivity analyses were conducted to evaluate whether individual studies influenced pooled ORs or HR by excluding one study by turns. The sensitivity analysis indicated that no study substantially influenced pooled OR/HR. This shifted effect measured of all studies and clinicopathological features/OS slightly, but did not change the significance level for any outcome, which confirmed the stability of meta-analyses.

### Subgroup analysis

Subgroup analysis was mainly performed on sample size to explored the potential sources of heterogeneity. Subgroup analysis on detection method was also used to explore the potential sources of heterogeneity of TNM stage.

As seen in Table 4, sample size didn't influence the relationship between Gli-1 expression and tumor location (*n* ≤ 70: OR 0.39, 95%CI 0.04-3.44, *P* = 0.393; *n* > 70: OR 0.82, 95%CI 0.42-1.61, *P* = 0.568). However, the heterogeneity of tumor location mainly existed in the small sample size subgroup (*n* ≤ 70) (*I^2^* = 83.6%, *P_bias_* = 0.014). Additionally, sample size didn't influence the relationship between Gli-1 expression and tumor size neither. And there were great heterogeneity in both subgroups (*n* ≤ 70: *I^2^* = 64.6%, *P_bias_* = 0.093; *n* > 70: *I^2^* = 80.0%, *P_bias_* = 0.025). While in both subgroups divided by sample size, Gli-1 expression was correlated to high depth of invasion in gastric cancer patients (*n* ≤ 70: OR 6.92, 95%CI 1.72-27.88, *P* = 0.007; *n* > 70: OR 9.24, 95%CI 2.85-29.92, *P* = 0.000). However, there was great heterogeneity in both subgroups (*n* ≤ 70: *I^2^* = 51.9%, *P_bias_* = 0.125; *n* > 70: *I^2^* = 71.7%, *P_bias_* = 0.029). Subgroup analysis by Gli-1 detection methods explored that high Gli-1 expression status was related to high TNM stage in IHC group (OR 4.86, 95%CI 2.67-8.82, *P* = 0.000), but not in ISH group (OR 0.64, 95%CI 0.16-2.55, *P* = 0.530). However, heterogeneity test showed that there was relatively low heterogeneity in IHC group (*I^2^* = 47.1%, *P_bias_* = 0.079), while no heterogeneity in ISH group (*I^2^* = 0.0%, *P_bias_* = 0.529). When divided by sample size, the subgroup analysis showed that in small sample size group (*n* ≤ 70), there was no relationship between high TNM stage and Gli-1 expression (OR 2.44, 95%CI 0.93-6.40, *P* = 0.069). While in bigger sample size group (*n* > 70), upregulated Gli-1was associated with high TNM stage (OR 5.33, 95%CI 2.21-12.85, *P* = 0.000). Nevertheless, there was great heterogeneity in both subgroups divided by sample size (*n* ≤ 70: *I^2^* = 53.1%, *P_bias_* = 0.074; *n* > 70: *I^2^* = 61.4%, *P_bias_* = 0.051). With these results, the heterogeneity of TNM stage was mainly caused by the different detection methods.

**Table 4:** Subgroup analysis of tumor location, tumor size, depth of invasion and TNM stage

Subgroups	Studies		OR(95%CI)	*z*	*P*_OR_	*I*^2^	*P*_bias_
**Tumor location**							
Sample size							
*n*≤70	2	0.39 (0.04, 3.44)		0.85	0.393	83.6%	0.014
*n*>70	2	0.82 (0.42, 1.61)		0.57	0.568	0.0%	0.387
**Tumor size**							
Sample size							
*n*≤70	2	0.90 (0.24, 3.35)		0.15	0.878	64.6%	0.093
*n*>70	2	2.93 (0.68, 12.72)		1.44	0.151	80.0%	0.025
**Depth of invasion**							
Sample size							
*n*≤70	3	6.92 (1.72, 27.88)		2.72	0.007	51.9%	0.125
*n*>70	3	9.24 (2.85, 29.92)		3.71	0.000	71.7%	0.029
**TNM stage**							
Detection method							
IHC	7	4.86 (2.67, 8.82)		5.19	0.000	47.1%	0.079
ISH	2	0.64 (0.16, 2.55)		0.63	0.530	0.0%	0.529
Sample size							
*n*≤70	5	2.44 (0.93, 6.40)		1.82	0.069	53.1%	0.074
*n*>70	4	5.33 (2.21, 12.85)		3.73	0.000	61.4%	0.051

Abbreviations: OR= odds ratio, CI = confidence interval, *n* = number of sample size, IHC = immunological histological chemistry, ISH = *in situ* hybridization.

## DISCUSSION

The Hh signaling pathway is considered to have a dominate role in tumorigenesis [6]. Recent studies found that the Hh signaling pathway could be abnormally activated in liver, bladder, and pancreatic cancer [26-28]. Gli-1, as one of Glis transcription factor family, has been identified as a marker of the aberrant activation of the Hh signaling pathway [29], it can stimulate the downstream target genes of Hh, such as Shh, Pintallavis/HNF-3β and N-tubulin [9]. This indicated that Gli-1 probably presented a vital function in tumorigenesis and tumor invasion. However, from the clinical perspective, a persuasive support of Gli-1′s clinical significance is still unavailable. A meta-analysis which combined a wide spectrum of carcinomas demonstrated the correlation between Gli-1 expression and poor prognosis [30]. However, this diversity between different carcinomas needs more consideration. Considering that one single study might be unconvincing, we performed the current meta-analysis to reach a reasonable conclusion.

To our knowledge, the present study is the first and most full-scale meta-analysis systematically explored the correlation between Gli-1 and clinicopathological features and prognosis in gastric cancer. Twelve eligible studies were summarized quantitatively based on our inclusion and quality assessment criteria. The meta-analysis indicates that Gli-1 is overexpressed in tumor tissue in comparison with the normal tissue, which is consisted with other studies [31-32]. What's more, our meta-analysis indicates that Gli-1 overexpression is correlated to poor differentiation type, high depth of invasion, lymph node metastasis and high TNM stage. To sum up, Gli-1 has a significant impact on neoplasm invasiveness-associated features. Several studies have found that Gli-1 can induce the expression of Snail [11]. As one of important transcriptional regulators of EMT, Snail can downregulate the expression of E-cadherin, as well as increase the expression of N-cadherin [10-11], which can contribute epithelial polarized cells turning into motile mesenchymal-appearing cells. Our results also affirm this mainstream viewpoint. It is worth noting that Gli-1 overexpression has impact on 5-year OS based on three related studies. However, in regard to the small number of studies included this meta-analysis, this standpoint needs further verification by incorporating more survival-related studies in future. However, there is significant heterogeneity in analysis of Gli-1 and several clinicopathological features, thus random effects model was chosen to determine pooled ORs.

Apart from the inspiring outcomes, some limitations still lay in this quantitative meta-analysis. Firstly, in this study, most of the Gli-1 expression in the included studies was measured by IHC method, therefore different primary antibody or different antibody concentrations could cause inconsistent Gli-1 detection. Secondly, the varied definition of cut-off values among the studies could also lead to potential bias. However, we were not able to conduct subgroup analysis by diverse antibodies or cut-off values due to small number of studies. Finally, the limited geographical area makes it difficult to indicate the relationship between Gli-1 and clinical features or prognosis among Western patients.

In spite of the limitations mentioned above, there are still numerous valuable implications of this comprehensive meta-analysis. On the whole, our results provide convincing proof of the correlation between aggressive biological behavior and Gli-1 overexpression in gastric cancer patients for the first time. Secondly, Gli-1 can be identified as a biomarker for poor prognosis in gastric cancer patients, which provides more guidance for clinical diagnosis and prognosis of these patients.

## MATERIALS AND METHODS

### Literature search strategy

This meta-analysis was conducted in accordance with the PRISMA guidelines. The Chinese databases of Wan Fang, China National Knowledge Infrastructure (CNKI) and Chinese VIP as well as English databases of Pubmed, Embase, and the Cochrane Library were retrieved from inception to April 28, 2016, using combinations of the following keywords: (“Gli-1” OR “Gli-1 protein, human [MeSH]” OR “Glioma-associated oncogene-1”) AND (“gastric carcinoma” OR “stomach cancer” OR “stomach neoplasms” OR “gastric cancer”). Additional relevant search was performed by manually searching the references of eligible studies or relevant reviews.

### Study selection criteria

Two independent investigators screened eligible studies by the same multistep procedures. First, investigators reviewed the titles and abstracts of the identified literature prudently. Studies which explored the relationship between Gli-1 expression and clinicopathological features or prognosis in gastric cancer patients were deemed to be eligible. Second, full texts of the eligible literatures were carefully reviewed and assessed according to the following inclusion criteria: (1) the study was published in English or Chinese with the full text available, (2) the study could be either randomized controlled study (RCT) or observational study (case-control or cohort), (3) the diagnosis of gastric cancer was confirmed using pathological examination, (4) Gli-1 expression was evaluated and based on primary gastric cancer tissue (neither serum nor any other kinds of specimen), (5) the study could provide sufficient information on the overall survival (OS) or clinicopathological indicators of patients related to the Gli-1 expression. Reviews, case reports, letters or animal studies were excluded.

### Data extraction and quality assessment

Two observers separately selected the eligible studies. Disagreements were settled by discussion with a third author if consensus could not be achieved by two observers. The following data were extracted from the eligible studies: the name of the first author, year of publication, country, and number of cases, detection methods of Gli-1 expression, antibody dilution, cut-off value of Gli-1, positive percentage, clinicopathological features, and the related survival data. Hazard ratio (HR) with 95% confidence interval (CI) of 5-year OS from univariate analysis was taken to count pooled HR. Calculation method introduced by Tierney et al [33] and Parmar et al [34] was applied to extract HR with 95%CI where HR was not reported. Kaplan-Meier (K-M) curves of those studies were read by Engauge Digitizer (version 4.1, http://digitizer.sourceforge.net/).

The quality of included studies was assessed by the Newcastle-Ottawa-Scale (NOS) criteria, and the study with NOS score of was 6 or higher was defined as a high-quality study, while the study with 5 or less score was considered as low-quality study.

### Statistical analysis

STATA version 12.0 was used to conduct all the statistical calculations. Pooled odds ratios (ORs) with 95% CIs were calculated to evaluate the association between positive Gli-1 expression and clinicopathological features (gender (male *vs*. female), tumor location (antrum *vs*. non-antrum), tumor size (≥ 5cm *vs*. < 5cm), differentiation type (poor/undifferentiated *vs*. well/moderate), depth of invasion (T3/T4 *vs*. T1/T2), lymph node metastasis (Yes *vs*. No), TNM stage (III/IV *vs*. I/II)), meanwhile, the difference of expression rate between cancer tissues and normal gastric tissues was also evaluated. And pooled HR with 95%CI was calculated to evaluate the prognostic significance of Gli-1 expression. *I^2^* test and *Q* test were used to assess heterogeneity among the studies. Fixed effects model was chosen preferentially when there was no significant heterogeneity. If heterogeneity was significant (*P_bias_* < 0.05), the random effects model would be used. The potential publication bias was examined by the funnel plots and Egger's tests. Sensitivity analysis was performed to investigate the source of heterogeneity and stability of results. Subgroup analysis was also conducted to explore the source of heterogeneity. Above all, the effects of Gli-1 expression on clinicopathological features and survival were considered as statistically significant if pooled estimates of OR/HR with 95% CI didn't overlap the value of 1. *P* values were two-sided, and the difference was considered as statistically significant when *P* < 0.05.
